# Mucinous Adenocarcinoma Arising in Chronic Perianal Fistula: Good Results with Neoadjuvant Chemoradiotherapy Followed by Surgery

**DOI:** 10.1155/2014/386150

**Published:** 2014-11-18

**Authors:** Marisa D. Santos, Carlos Nogueira, Carlos Lopes

**Affiliations:** ^1^Departamento de Cirurgia, Serviço de Cirurgia Digestiva, Hospital de Santo António, Largo Professor Abel Salazar, 4099-003 Porto, Portugal; ^2^Departamento de Patologia, Serviço de Anatomia Patológica, Hospital de Santo António, Largo Professor Abel Salazar, 4099-003 Porto, Portugal; ^3^Departamento de Patologia e Imunologia Molecular, Instituto de Ciências Biomédicas Abel Salazar, Rua Jorge Viterbo Ferreira No. 228, 4050-313 Porto, Portugal

## Abstract

Chronic perianal fistulas are a common clinical condition. However, their evolution to adenocarcinoma is rare. We report the case of a 48-year-old man with perianal chronic fistulas, who developed two perianal ulcerated lesions near the external orifices of the fistulas, which extended proximally as a pararectal tumor. No intestinal lesion was seen at endoscopic examination. Histopathological biopsy indicated mucinous adenocarcinoma. Staging was performed by pelvic magnetic resonance imaging (MRI) and thoracoabdominal CT scan. The patient underwent a laparoscopic colostomy followed by neoadjuvant chemoradiotherapy and then laparoscopic abdominoperineal resection followed by adjuvant therapy. We have seen a favorable outcome with no recurrence at 3 years of follow-up.

## 1. Introduction

Perianal mucinous adenocarcinoma is a rare disease often associated with a long-standing anal fistula, representing approximately 2-3% of large bowel cancers [1–4]. The occurrence of a carcinoma arising in a fistula is probably due to chronic inflammation [3]. Detection is usually late as the symptoms often initially mimic benign inflammatory conditions of the anorectal region and biopsies fail to reveal the infiltrating carcinoma [5, 6]. The diagnosis is challenging and is based on a high index of clinical suspicion and specific histologic features [3, 7]. Due to the rarity of this tumor and the lack of sufficient patients for controlled trials, there is no consensus regarding diagnosis and treatment strategies.

We report a rare case of large mucinous adenocarcinoma, arising from a perianal fistula, successfully resected after neoadjuvant chemoradiotherapy.

## 2. Case Report

**Figure 1:**
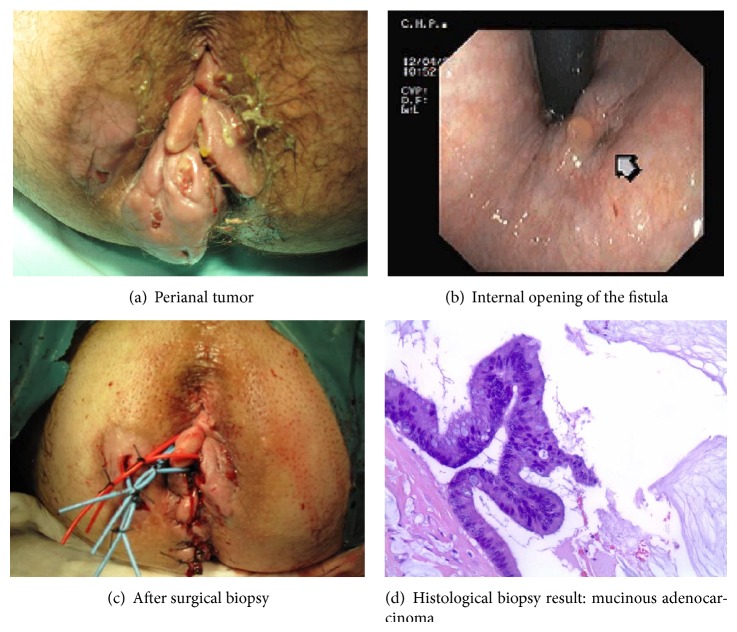
The diagnosis.

**Figure 2:**
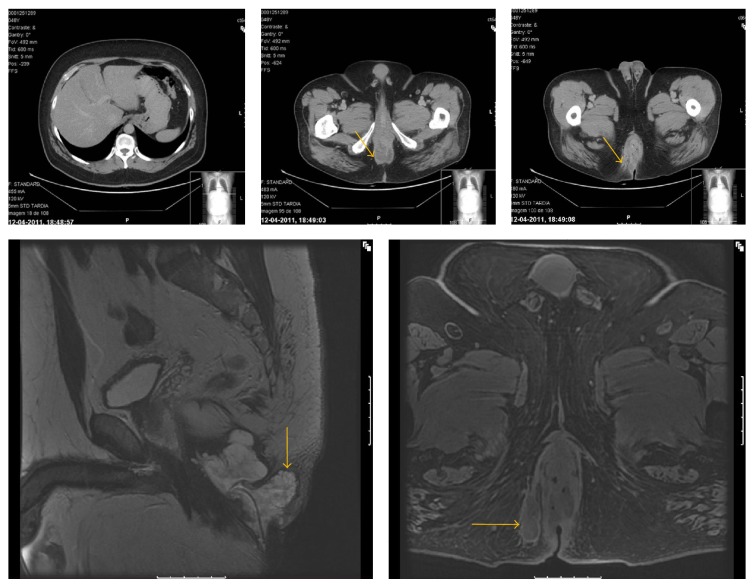
Tumor staging.

A 48-year-old man with a 12-year history of perianal fistula who had recently been suffering from mucinous discharge, pain, and perianal induration was referred to hospital for treatment. One year before, he had undergone urgent surgery for incision and drainage of recurrent perianal abscess. Physical examination revealed two indurated, ulcerative lesions with 7 cm in diameter with three external anal fistula openings (Figure 1(a)), without enlarged inguinal lymph nodes. No lesion was found on upper endoscopy. On colonoscopy, the internal opening of the fistula was indicated at the right side of dentate line and there was no tumor in the rectum on digital examination or any evidence of mucosal lesion in the colon or rectum (Figure 1(b)). CT scan and magnetic resonance imaging (MRI) demonstrated a large demarcated tumor at the level of the anorectal junction with extension to the right side of the ischiorectal fossa (Figure 2). Enlarged pelvic lymph nodes were observed and there was no evidence of inguinal nodal involvement or distant metastasis (Figure 2). Histological examination of a biopsy specimen taken from the induration revealed mucinous adenocarcinoma (Figures 1(c) and 1(d)). Laboratory data showed a normal carcinoembryonic antigen serum level. Before initiating therapy a laparoscopic colostomy was performed to control the suppurative process. To decrease the rate of local recurrence, neoadjuvant chemoradiotherapy was given: a total irradiation of 50.4 Gy in 28 fractions and 5-fluorouracil (5-FU) by infusion pump. Eight weeks after radiotherapy the patient underwent radical resection.

## 3. Operative Technique Details

A conventional laparoscopic abdominoperineal resection (APR) in a lithotomy position was performed.

The abdomen and perineum were prepared and draped and the anus was closed with stout purse-string suture.

Carbon dioxide pneumoperitoneum to a pressure of 12 mm Hg is achieved using a closed technique. A 10 mm trocar is inserted whereupon the 10 mm telescope is introduced. A 5 mm trocar in upper right quadrant, a 12 mm trocar in the lower quadrant, and a 5 mm suprapubic port are placed. Preliminary laparoscopic inspection was performed.

We elevate and put traction of the sigmoid, identifying the inferior mesenteric pedicle. We dissect the retroperitoneal space with visualization of the left ureter. We proceed with ligation and division of inferior mesenteric artery and vein. Then we mobilize the rectum by carrying dissection into the avascular areolar tissue of the presacral plane, in accordance with the principles of TME. Meticulous dissection of the mesorectum from the presacral fascia was continued to the tip of the coccyx, taking care of not breaching the interface between the tumor and the elevators. Mesocolon distal to colostomy was ligated and descendent colon was transected with Endo-GIA help.

Attention was then turned to the perineal phase of the procedure. The skin surrounding the tumor was excised with adequate tumor-free margin. Dissection was performed in the extrasphincteric plane with the pelvic cavity being entered just anterior to the coccyx. Division of the elevators was then undertaken from posterior to anterior. The dissection remaining near from external anal sphincter but including tracts and extent tumor in ischiorectal fossa. Following dissection of the specimen from the anterior structures, the specimen was removed through the perineal wound.

Primary closure of the perineum was performed by approximating the residual elevator musculature by using sutures, without need to reconstruct the pelvic floor with a rectus abdominis or gracilis muscle flap.

**Figure 3:**
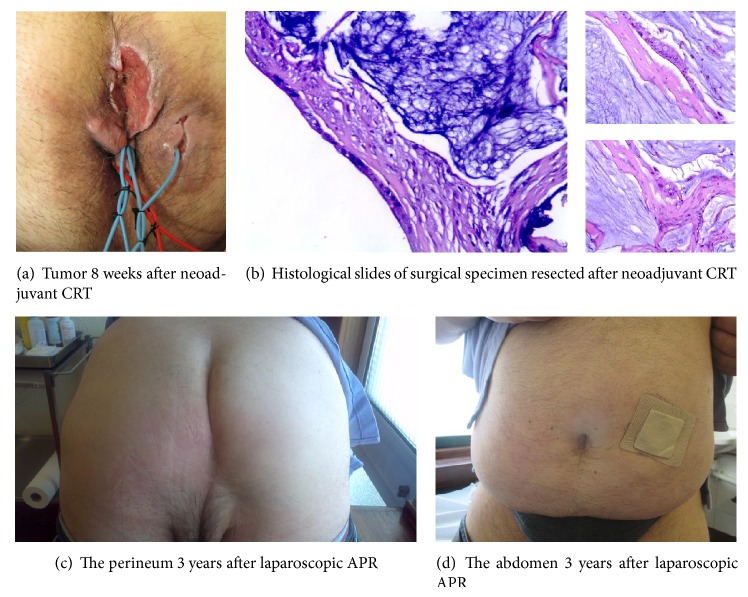
Treatment results.

The postoperative course was uneventful and the patient discharge from hospital was 5 days after surgery. The resected specimen revealed the tumor developed in the ischiorectal fossa with extension to anal sphincters, but there was no evidence of cancer extension to the mucosal surface of the anal canal and rectum. Histological examination of the excised specimen showed foci of mucinous adenocarcinoma involving the lower internal and external anal sphincter muscles, and the resected margins did not show any residual tumor (Figure 3(b)). According to the UICC TNM staging criteria, the treatment outcome with preoperative chemoradiotherapy followed by surgery revealed moderate response (grade 1) (Figure 3(b)). Metastases to the lymph node were not detected (0/16). After the operation, the patient was subjected to adjuvant chemotherapy for 6 months performed with 5-FU.

Three years later the patient has no evidence of disease and both the functional and cosmetic surgical results are good (Figures 3(c) and 3(d)).

## 4. Discussion

The development of a perianal mucinous adenocarcinoma arising in long-standing perianal fistulas is rare, with few reports in the literature [1, 3, 4, 8, 9]. The pathogenesis, biological behavior, and treatment of this disease remain controversial [1–4, 8]. Many authors have favored an origin in the anal glands. Another theory is that deposition of malignant cells in the granulation tissue of a fistula arises from a proximal gastrointestinal cancer. It is necessary, therefore, to evaluate the entire gastrointestinal tract before assuming that the carcinoma is primary to the fistula. The presence of a long history of fistula-in-ano and the exclusion of another carcinoma allow the diagnosis of carcinoma arising in a benign fistula, as in our case. The required duration of symptoms of the fistula was arbitrarily set at 10 years [4]. Crohn's disease, tuberculosis, syphilis, and lymphogranuloma venereum were considered in the differential diagnosis. Although the diagnosis can and should be suspected on clinical grounds, such as sudden lesion growth or the appearance of a new mucoid instead of purulent tumor discharge, it can be established only by intraoperative biopsy, requiring general anesthesia in this case. The presence of mucinous material led to the diagnosis (Figure 1(d)). Colonoscopy, TC scan, and magnetic resonance imaging confirmed the diagnosis and allowed the disease staging.

Mucinous adenocarcinoma in a long-standing fistula-in-ano is known to be a slow growing, locally aggressive neoplasm with a low-grade histologic appearance and rarity of metastasis [2]. The standard treatment option for these patients has been surgical. Abdominoperineal resection is the most frequently employed operation [3, 8, 10].

Tumor spread is usually lymphatic, and the inguinal lymph nodes are the most frequent sites of metastasis. Like the majority of patients in previous reports, our patient had extensive disease at the time of presentation and pelvic, but no inguinal nodal involvement. These tumor aspects are usually related to decreased survival. In the present case, total removal of the tumor without any residual disease was quite difficult because of the size of the tumor and the fact that it had infiltrated the surrounding tissue. For this reason we approached this case like a locally advanced rectal cancer (LARC), neoadjuvant chemoradiotherapy followed by surgery and adjuvant chemotherapy, although the role of CRT in the treatment of perianal mucinous adenocarcinoma has not yet been established [1, 2, 10]. There are not enough reported cases undergoing this therapy to determine the prognosis for individual patients. In our case the neoadjuvant CRT caused tumor regression and downstaging (Figure 3(a)): this fact allowed us a laparoscopic AbdominoPerineal Resection (APR) instead of ExtraLevatory AbdominoPerineal Excision (ELAPE) wich may lead to the reconstruction of the pelvic floor with mesh or muscle flap, probably increasing short-term complications [11]. We believe that neoadjuvant chemoradiotherapy plays an important role in the treatment of locally advanced disease and in this case contributed to the good result obtained.

In conclusion, fistula-associated anal mucinous adenocarcinoma is an uncommon complication of chronic perianal fistula. The diagnosis is often unsuspected. Thus, tissue from chronic anal fistula tracts should be submitted for pathologic evaluation. The result of this case suggests that neoadjuvant chemoradiotherapy followed by surgery and adjuvant chemotherapy appears to be a valuable alternative treatment for patients with perianal mucinous adenocarcinoma arising from a perianal fistula. The advantage of this approach can result in downstaging and R0 resection, which prevents local recurrence.
